# Characterizing biobank organizations in the U.S.: results from a national survey

**DOI:** 10.1186/gm407

**Published:** 2013-01-25

**Authors:** Gail E Henderson, R Jean Cadigan, Teresa P Edwards, Ian Conlon, Anders G Nelson, James P Evans, Arlene M Davis, Catherine Zimmer, Bryan J Weiner

**Affiliations:** 1 Department of Social Medicine, CB 7240, University of North Carolina at Chapel Hill, Chapel Hill, NC 27599-7240, USA; 2 HW Odum Institute for Research in Social Science, University of North Carolina at Chapel Hill, Chapel Hill, NC 27599-3355, USA; 3 Department of Sociology, University of North Carolina at Chapel Hill, Chapel Hill, NC 27599-3355, USA; 4 Department of Genetics, CB 7264, University of North Carolina at Chapel Hill, Chapel Hill, NC 27599-7264, USA; 5 Department of Health Policy and Management, CB 7411, UNC Gillings School of Global Public Health, University of North Carolina at Chapel Hill, Chapel Hill, NC 27599-7411, USA

**Keywords:** Biobank, biorepository, governance, survey

## Abstract

**Background:**

Effective translational biomedical research hinges on the operation of 'biobanks,' repositories that assemble, store, and manage collections of human specimens and related data. Some are established intentionally to address particular research needs; many, however, have arisen opportunistically, in a variety of settings and with a variety of expectations regarding their functions and longevity. Despite their rising prominence, little is known about how biobanks are organized and function beyond simple classification systems (government, academia, industry).

**Methods:**

In 2012, we conducted the first national survey of biobanks in the U.S., collecting information on their origins, specimen collections, organizational structures, and market contexts and sustainability. From a list of 636 biobanks assembled through a multi-faceted search strategy, representatives from 456 U.S. biobanks were successfully recruited for a 30-minute online survey (72% response rate). Both closed and open-ended responses were analyzed using descriptive statistics.

**Results:**

While nearly two-thirds of biobanks were established within the last decade, 17% have been in existence for over 20 years. Fifty-three percent listed research on a particular disease as the most important reason for establishment; 29% listed research generally. Other reasons included response to a grant or gift, and intent to centralize, integrate, or harmonize existing research structures. Biobank collections are extraordinarily diverse in number and types of specimens and in sources (often multiple) from which they are obtained, including from individuals, clinics or hospitals, public health programs, and research studies. Forty-four percent of biobanks store pediatric specimens, and 36% include postmortem specimens. Most biobanks are affiliated in one or multiple ways with other entities: 88% are part of at least one or more larger organizations (67% of these are academic, 23% hospitals, 13% research institutes). The majority of biobanks seem to fill a particular 'niche' within a larger organization or research area; a minority are concerned about competition for services, although many are worried about underutilization of specimens and long-term funding.

**Conclusions:**

Effective utilization of biobank collections and effective policies to govern their use will require understanding of the immense diversity found in organizational features, including the very different history and primary goals that many biobanks have.

## Background

Biobanks are repositories that assemble, store, and manage collections of human specimens and related data. Although they have existed in some form for over 60 years, their recent surge in numbers, size, and prominence [1] has focused attention on the changing nature of biomedical research and relationships among investigators, research participants, and the organizations that fund and manage these entities [2-9]. This surge in numbers coincides with success in sequencing the human genome in 2003, the subsequent explosion of new bioinformatics technologies and developments in next-generation sequencing [10,11], and the vision of improved health through genomic medicine [12]. In short, effective translational biomedical research discoveries depend upon the operation of biobanks.

Despite this reliance, we know little about biobanks. The first attempt to catalog human tissue sources in the U.S. was undertaken by the RAND Corporation [13], coinciding with the first National Bioethics Advisory Commission report on ethical and policy issues involving human biological materials [14]. Through its *Handbook *and the 12 case studies conducted four years later [15], RAND provided information on specimen existence, location, and access, noting that not all forms of repositories were captured. The legacy of the RAND work is the classification of biobanks into three categories (government, academia, industry) that persists in the literature [15]. Notwithstanding recent interest in classifying biobanks [5,16], there is little empirical research on the organization and functioning of biobanks that might inform a more nuanced, updated classification system.

There are several reasons for this dearth in empirical research. Significant variation in biobank types leaves scholars unable to agree upon a definition [5,17-25]. In addition, no census or registry of biobanks exists from which to draw representative samples for study. Thus, while many authors have observed the importance of organizational context for understanding biobank policies, it is understudied empirically [5,8,9,19,26-28]. In fact, most empirical studies relevant to policy issues (for example, informed consent, identifiability, return of research results) have focused not on biobanks, but on the views of external stakeholders [29-38].

To address this gap in knowledge, we sought to describe U.S. biobanks in terms of their origins, specimen collections, organizational features, and market contexts. We applied a systematic, multi-faceted strategy to develop a list of biobanks in the U.S., and fielded a survey. Our findings document extraordinary diversity, and present baseline data upon which future analyses of organizational change, policy, and regulatory frameworks may rest.

## Methods

The U.S. Biobank Survey is part of a larger study of biobanks in the U.S., funded from 2010 to 2012 by grants from the National Human Genome Research Institute and a supplement to the University of North Carolina (UNC) Clinical and Translational Science Award (CTSA). The objectives were to examine the diversity of biobanks' organizational characteristics, policies, and practices. For the purposes of our study, a biobank was defined as 'an organization that acquires and stores human specimens and associated data for future research use.' Our work included conducting six qualitative case studies, constructing a list of biobanks in the U.S. [39], and piloting and administering a 30-minute online survey. We collected data from U.S. biobank representatives on how their biobanks operate, components of organizational features identified in the literature, and the policies and practices biobanks have in place that govern their relationships with the individuals who contribute specimens and researchers who use them. This study was approved by the UNC Institutional Review Board.

### Construction of the list of U.S. biobanks

Because there is no generally agreed upon definition of a biobank and no reliable census of these heterogeneous entities in the U.S., it is not possible to draw a representative sample for a national survey. In an attempt to create a comprehensive list of U.S. organizations fitting our definition of a biobank, we cast a wide net [39]. We employed multiple search strategies, similar to those used by RAND [13]. We searched articles listed in PubMed for the year 2010 and current federally awarded grant abstracts from National Institutes of Health (NIH)'s RePORTER database; and performed a systematic Google search consisting of two parts - an initial search that found individual biobanks and a follow-up search of lists and directories of biobanks that appeared in the initial search. In addition, because a significant portion of medical research is sponsored by medical institutions and research institutes, and more recently by NIH-sponsored CTSAs, we conducted searches on American Association of Medical Colleges member websites and on CTSA websites. Lastly, similar to RAND, we communicated with other investigators involved in biobank research to provide additional names, and in one case, obtained a list of biobanks [28].

We used a wide variety of keywords in our multi-faceted online searches, constructing nested Boolean strings to account for the fact that some terms imply the storage of specimens (for example, 'biorepository') while other terms do not (for example, 'collection') [39]. Biobanks that did not aim to share specimens were excluded, as were biobanks without some focus on domestic research. If available information was ambiguous, we contacted the organization for clarification.

We carefully recorded and cross-referenced all sources so that we could both detect duplicates and identify the strengths and weaknesses of different sources. Of necessity, we restricted our focus to organizations for which we could find at least minimal presence on the web. That presence ranged from active marketing of the collection, to being named as entities in academic institutions or independent organizations where investigators store research specimens, to being described in an organizational newsletter.

**Figure 1:**
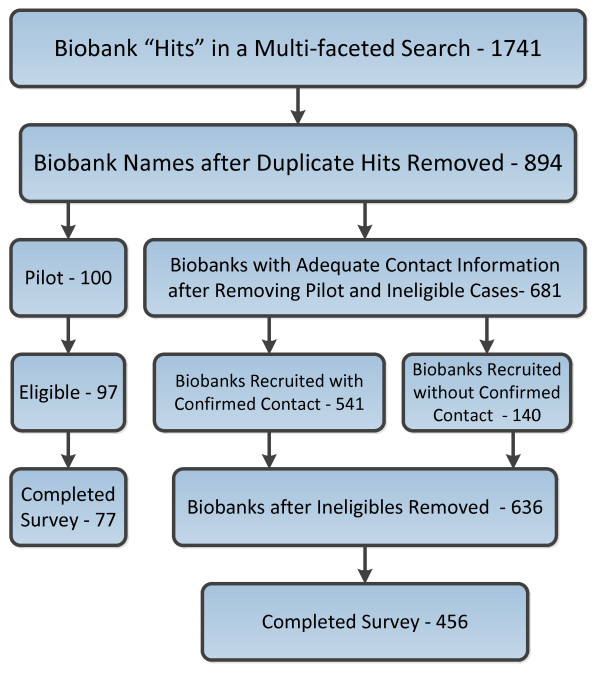
**Number of biobank names initially identified, and process of determining eligibility for survey recruitment**.

Figure 1 depicts the number of biobank names initially identified, and the process by which we ultimately produced a list for survey recruitment. By eliminating duplicates, we reduced our initial 1,741 names of possible biobanks to 894. We then endeavored to identify the name and current email and postal mail addresses for the director, manager, or other representative from each biobank whom we would eventually recruit to take the survey. We contacted, through email or telephone, these representatives to confirm they had the appropriate knowledge about the biobank to take our survey. In many cases they did, but in some instances the person we contacted nominated someone else in the organization. We attempted to confirm the contact information of the nominated individual. During this process, we discovered entities that did not fit our definition of a biobank, were no longer in existence, or whose collections had been transferred to another biobank; we marked these cases as ineligible. Finally, there were some biobanks for which we were never able to discern adequate contact information (either an email address or telephone number), so we were forced to drop them from our list of 894. Ultimately, we identified biobanks in 43 states plus the District of Columbia.

### Survey development and pilot

Questions for the survey were based on results of our six case studies, prior research studies (including surveys of other types of organizations), and the organizational sociology literature. We partnered with the UNC Odum Institute for Research in Social Science for methodological guidance on questionnaire design and on all aspects of survey data collection. Given the measurement challenges we faced, we devoted a six-month period in 2011 to pilot testing all aspects of our survey plan.

For the pilot, we chose a purposive (non-random) sample of 100 biobanks from our list, ensuring that there was representation of the four types of URL extensions in our list (.com, .edu, .gov, and .net, or .org). We first notified biobanks about the upcoming survey via a FedEx letter explaining the study and the incentive for completion (a $30 Amazon gift card). As described above, prior to sending the letters, we emailed all biobanks selected for the pilot and asked them to confirm that we had the correct name and contact information for a representative with the knowledge to complete the survey. When necessary, we followed up with phone calls to verify these details. Using such thorough procedures, we were confident we were contacting the correct individual and were able to customize the recruitment letters with the recipient's name. Our goal was to get the potential respondent's attention in a professional way that motivated them to watch for the survey invitation in their email inbox and to complete the survey promptly.

Approximately four days after the FedEx letters were delivered, we emailed invitations to the identified respondent at each biobank. The email contained a hyperlink which took the respondent directly to the survey. Of the 100 individuals we contacted, three replied that their organization did not meet our definition of a biobank and were therefore ineligible. Of the remaining 97 biobanks, 77 completed the survey, for a response rate of 79%. Results of the pilot study were used to revise and improve the survey instrument in preparation for our main survey.

### Survey of U.S. biobanks

Though we modified the survey questionnaire in response to our pilot data, we changed little else in our procedures for the main study. Excluding the 100 biobanks recruited for the pilot survey, our list included 681 biobanks (Figure 1). Of these, we had confirmed contact information for 541; as in the pilot study, these biobanks were recruited by letter sent via FedEx or USPS Express. We did not receive responses to our email and telephone requests to confirm their existence or contact information from140 biobanks in our list. Without a confirmed contact person or postal address, these biobanks were recruited via email using the email address we had found in our search. In February 2012, a few days after sending the recruitment letters and emails, we emailed survey invitations to these 681 banks. We used email reminders and phone calls to prompt nonrespondents. In the process of data collection, we discovered that an additional 45 biobanks (35 from the 'confirmed contact' group and 10 from the 'unconfirmed contact' group) were ineligible, thus producing a total of 636 eligible biobanks in our study. Upon conclusion of data collection in May 2012, we had 456 completed surveys, representing a response rate of 72%.

### Differential response rates and nonresponse bias

As might be expected, we achieved a significantly higher response rate among the biobanks with previously confirmed contact information than those without (81% versus 34%, *P *< 0.001). However, only 20% of biobanks (130 of 636) fell into the latter category, so our overall response rate remained high. Assessing risk of nonresponse bias requires observations on both responding and nonresponding units [40]. The only additional, potentially relevant information we have on nonresponding biobanks is the domain portion of their website URL (.com, .edu, .gov, and .net, or .org). We examined response rates by domain and found no statistically significant differences.

Table 1 shows the composition of the responding biobanks compared to the entire list. The responding biobanks closely correspond to the distribution among the four URL domain types.

**Table 1:** Comparison of responding biobanks to master list

	Responding biobanks		Master list	
	n	%	n	%
**Contact information confirmed prior to survey invitation?**				
Yes	413	91	507	80
No	43	9	130	20
Total	456	100	637	100
**Domain portion of URL**				
.com	26	6	40	6
.edu	296	65	399	63
.gov	26	6	32	5
.org or.net	108	24	165	26
Total	456	100	637	100

### Coding and analysis methods

Two types of open-ended questions were used in the survey. The first were stand-alone questions such as 'What is your title or role at [BIOBANK]?' The second were open-ended questions embedded in fixed-response questions, and were typically designed to allow respondents to offer information beyond the fixed responses. For example, we asked, 'What is the main biomolecule [BIOBANK] isolates from specimens, if any?' Fixed responses included 'DNA,' 'RNA,' 'Protein,' 'Other,' and 'None.' Respondents who chose 'Other' were given an open-ended text box to specify other biomolecules. A codebook was developed for each open-ended question by at least three of the authors (AGN, GEH, and RJC). All open-ended responses were coded in a systematic manner by a primary coder (AGN). Two secondary coders (GEH and RJC) reviewed all codes proposed by the primary coder. When the secondary coders did not agree with the primary coder's choice of codes, the three coders met and resolved inconsistencies through consensus.

Survey data were collected using Illume software version 4.7 (Datstat, Inc., Seattle, WA, USA). Data were analyzed in SAS version 9.2 (SAS Institute Inc., Cary, NC, USA). We present simple response frequencies and cross-tabulations, with percentages where appropriate. Where percentages do not add to 100, it is due to rounding. Most variables are discrete; thus we used chi-square for tests of statistical significance except when small cell sizes dictated use of Fischer's Exact Test.

## Results and discussion

### Origins of U.S. biobanks

Table 2 shows the reported dates of establishment for biobanks in our survey. Though over half (59%) were established since 2001, 17% have been in existence for more than 20 years, with seven banks over 50 years old.

**Table 2:** Year of establishment

	n	%
1980 or earlier	30	7
1981 to 1990	46	10
1991 to 2000	109	24
2001 to 2010	249	56
2011 or later	13	3
Total	447	100

Respondents were asked to 'check all that apply' in a list of reasons that their biobank might have been established, and then asked to report the most important reason. Table 3 shows that over half of the banks (53%) were established primarily to facilitate research on a particular disease or type of disease. Though we did not ask what specific disease prompted establishment, it is clear from reading responses to this and other open-ended survey questions that by far the largest portion focus on cancer, followed by neurological diseases (such as Alzheimer's), and HIV/AIDS. The second most frequent primary reason for establishment was to facilitate research generally (29%). The remaining 19% of biobanks were primarily developed for various unique reasons specific to their circumstances, such as in response to a grant, to organize or consolidate existing collections, or to store specimens for others.

**Table 3:** Primary reason for establishment by date

Year established	Facilitate research on a particular disease or type of disease		Facilitate research generally		Other reason		Total	
	n	%	n	%	n	%	n	%
Before 2003	137	59	50	22	45	19	232	54
2003 or later	87	45	73	38	34	18	194	46
Total	224	53	123	29	79	19	426	100

Χ^2 ^= 13.71, *P *< 0.01

As shown in Table 3, we examined whether biobanks established prior to the sequencing of the human genome in 2003 were more likely to have been established to facilitate research on a particular disease compared to those established after 2003 (59% versus 45%). After 2003, although studying a particular disease was still the most common reason for establishment, significantly more biobanks were created to facilitate research generally, compared to years prior to 2003 (38% versus 22%, *P *< 0.01). While there are likely multiple explanations for these results, it is possible that the changing landscape of genomic technology has facilitated a broadening of scope in research pursuits so that biobanks are not as likely to limit their work to one disease.

### Specimen collections

The number of specimens currently in storage by the responding biobanks ranged from tens to over 50 million. The distribution is shown in Table 4. Due to a small number of very large banks, the mean number of specimens reported was 461,396; the median was 8000. As can be seen from Table 4, it is difficult to discern a 'typical' sized collection; rather, biobank collections in the U.S. cover a wide spectrum of very small to very large. It should be noted that the survey question asked how many specimens the bank currently stored, which may not be the best measure of size for some banks. Some respondents provided comments at the end of the survey indicating that the number of specimens they have in storage at any given time varies greatly.

**Table 4:** Number of specimens in storage

	n	%
Less than 500	63	15
500 to 999	28	7
1000 to 1999	31	7
2000 to 4999	54	13
5000 to 9999	44	10
10,000 to 49,999	70	16
50,000 to 99,999	38	9
100,000 to 499,999	65	15
500,000 +	33	8
Total	426	100

Mean: 461,396; standard deviation: 3,324,096; median: 8000; interquartile range: 1,200 to 76,000; skewness: 12.76; kurtosis: 181.

The number of specimens in storage can reflect duplicate contributions and processed derivatives of contributed samples, so we also asked respondents approximately how many individual contributors were represented among their specimens in storage. Responses ranged from just a few to 10 million. To get an idea of the number of contributors typically represented in biobanks relative to the number of specimens, we calculated the ratio of specimens to contributors for each biobank. The values ranged from 1 (one specimen per contributor) to 277. The mean number was 12.6; however the distribution was skewed by a small number of banks with large numbers of specimens per contributor. Thus, the modal response was 1 and the median was 3.2 even though the mean was 12.6. Most biobanks (65%) had a ratio of 5 or fewer specimens to contributors. Thus, it appears that most biobanks contain only a small number of specimens from each contributor.

We asked respondents which type(s) of biological specimen(s) their bank stores. As shown in Table 5, serum or plasma are the specimens most commonly stored (77% of biobanks have them) with solid tissues following close behind (69%). Fifty-five percent of biobanks store whole blood, and 49% store peripheral blood cells or bone marrow. Though cord blood or cord derivatives were the least common among the categories we specifically asked about (11%), by coding the 'other, please specify' responses, we determined that 7% of biobanks store pathological body fluids (such as the peritoneal fluid that accumulates in ascites) and 3% store hair or toenails - two categories we had not anticipated to be this common.

**Table 5:** Types of specimens in storage (check all that apply)

Percentage of biobanks storing specimens of this type	n	%
Serum or plasma	349	77
Solid tissue specimens, including paraffin-embedded, frozen, or other	315	69
Whole blood	251	55
Peripheral blood cells or bone marrow	222	49
Cell lines	162	36
Saliva or buccal cells	155	34
Urine or stool	138	30
Cerebral spinal fluid	85	19
Cord blood or cord blood derivatives	51	11
Other biological specimens	40	9
Pathological body fluids	30	7
Hair/toenails	14	3

As shown in Table 6, most biobanks (87%) store more than one type of specimen; 8% store eight or more types. The most frequent combination of types was whole blood, plasma, and solid tissues. Banks with only one type of specimen are most likely to be those which only store solid tissue.

**Table 6:** Number of types of specimens in storage

	n	%
1	58	13
2	59	13
3	81	18
4	62	14
5	66	15
6	50	11
7	38	8
8+	36	8
Total	453	100

We asked respondents to identify the main biomolecule their bank isolates from specimens, if any. Nearly 50% said 'DNA,' 11% said 'RNA,' and 7% said 'Protein.' Twenty-four percent of respondents indicated 'None.' The 9% who chose 'Other' provided open-ended responses to explain. Virtually all indicated that they were unable to choose just one biomolecule because the biobank isolates more than one in equal proportions (most frequently DNA and others). The large percentage of respondents who indicated their banks were isolating DNA (and RNA) suggests that the majority of biobanks are engaging in genetic research of one sort or another.

We wanted to determine biobanks' sources for acquiring specimens. As shown in Table 7, the two largest sources of specimens are direct contribution by individuals (75%) and residual specimens from hospitals and other clinical settings (57%). In fact, many (41%) include specimens from both these sources, and only 8% do *not *report either individuals or clinical settings as sources of specimens. The third largest source of specimens is research studies (13%). Other sources reported by a small number of biobanks include vendors, organ or body donation organizations, other repositories, or that they acquired 'orphaned' collections (those which were presumably abandoned by their original owners).

**Table 7:** Acquisition of specimens (check all that apply)

**Percentage of biobanks which get specimens from**...	n	%
Direct from individuals donating them	343	75
Residual specimens acquired from clinical care in hospitals, clinical laboratories, or pathology departments	261	57
Research	60	13
Residual specimens from public health departments or programs	19	4
Vendors	8	2
Organ/body donation organization	7	2
Other repositories	7	2
Other	6	1
Orphaned collections	4	1

Respondents were asked whether specimens in their collection represent any particular group(s) of individuals. Forty-four percent of biobanks store specimens from children under the age of 18, though only 2% house exclusively pediatric specimens. In 36% of biobanks, the collection includes specimens collected postmortem, including 9% which store exclusively postmortem specimens, most of which are brain banks.

As shown in Table 8, 76% of respondents indicated their biobank's specimens come primarily from individuals with a particular disease or type of disease, and an equal proportion reported that their specimens come primarily from patients in a specific hospital or clinic. Over half (60%) endorsed both 'individuals with a particular disease or type of disease' and 'patients from specific hospital(s) or clinic(s).' It is not clear from our data whether this represents two descriptors of the same group (patients in particular clinical settings who have a particular disease) or whether these biobanks have different sub-collections, though we believe the former explanation is most logical. Similarly, 34% of respondents reported both that their biobank's specimens are primarily drawn from clinical trials patients and from specific hospital or clinical settings. An equal fraction (34%) endorsed both 'participants in clinical trials' and 'patients with a particular disease or type of disease.'

**Table 8:** Specimen contributors (check all that apply)

**Percentage of biobanks reporting that their specimens are drawn primarily from**...	n	%
Individuals with a particular disease or type of disease	335	76
Patients from specific hospital(s) or clinic(s)	331	76
Participants in a cohort study	184	40
Participants in clinical trials	180	39
Individuals from a specific geographic area (including community-based biobank)	122	27

### Organizational characteristics

Only 5% of responding biobanks are for-profit organizations. Seven percent are incorporated. Regardless of organizational form, 80% have internal oversight boards of some kind. We asked whether each biobank is 'part of a network of biobanks.' Sixteen percent indicated they were and then filled in the name of the network. Based on detailed review of these open-ended responses, it is clear that respondents interpreted 'network' broadly. Responses included programs, registries, cooperative groups, multi-site studies, and consortia that were NIH sponsored, state-sponsored and population based, intra- and inter-institutional, and national and international. A few banks listed more than one network.

Most biobanks are affiliated in one or multiple ways with other organizations. Determining the presence and nature of biobanks' affiliations or locations within larger organizations proved very difficult - so difficult, in fact, that it took us three attempts to develop a suitable question. Following the pilot study, we rewrote the survey questions about being part of a larger organization. Despite our best efforts, it became clear midway through our data collection effort that we still had not asked the questions in a way respondents understood as we intended. For example, we found biobanks that were clearly part of universities whose respondents identified the bank as an 'independent organization.' For this reason, we clarified the wording of our survey question (adding 'such as a university, a hospital, government, a research institute, or any other type of larger organization' to our original question wording of 'Is your biobank an independent organization or part of some larger organization?'). We re-contacted the 121 biobanks who had responded that they were 'independent' prior to this modification in order to ensure they were properly classified. Through these efforts, we determined that 88% of responding biobanks were part of larger organizations and only 12% were independent in the ways we intended to use the terms.

Further characterizations of biobanks within larger entities were equally challenging. When biobanks were part of larger organizations, we asked respondents to indicate the nature of the larger organization(s), using a provided list of eight organizational types and space to write in others. Among biobanks that are part of one or more larger organizations (which we call 'embedded'), Table 9 shows the percentage that checked each type of organization. Clearly the most frequent affiliation of a biobank is within an academic institution (78% of embedded banks). Hospitals were reported as a parent organization for 27%, and 15% were part of a research institute. About a quarter (28%) of all biobanks are part of more than one larger organization. Table 10 provides details. By far the most common situation of multiple affiliation is for a biobank to be affiliated with an academic institution and also with another organization - a hospital being a second affiliation for more than half (73%) of academic biobanks with multiple affiliations. The next most common multiple affiliation is within an academic institution and also a research institute (34% of biobanks affiliated with academic institutions are also part of a research institute).

**Table 9:** Type of larger organizations biobank is part of (check all that apply)

	n	%
Academic institution	307	78
Hospital or health care organization	105	27
Research institute	60	15
Federal government	38	10
Disease or health advocacy organization	19	5
State government	17	4
Philanthropic organization	11	3
Corporation	10	3
Other	7	2
Consortium	5	1

Note: Table includes only biobanks which are embedded in at least one larger organization (*n *= 394)

**Table 10:** Number of types of organizations biobank is part of, by academic affiliation

	With academic affiliation		Without academic affiliation	
	n	%	n	%
0 (independent)	0	0	54	38
1	189	62	77	55
2	71	23	10	7
3	38	12	0	0
4	5	2	0	0
5	3	1	0	0
6	1	0	0	0
Total	307	100	141	100

Very few non-academic biobanks are embedded within more than a single larger organization. Most are solely within a single larger entity, and it is most likely to be the federal government (29%), or a hospital or research institute (18% each).

For many biobanks, being embedded within a larger organization is critical to its financial structure. Together with the federal government, the larger organizations named by biobanks are the most common and largest sources of funding. Tables 11 and 12 show the results of our questions regarding the bank's largest source of current funding (Table 11) and funding provided within the past 5 years (or since establishment for biobanks younger than 5 years) by a list of possible sources (Table 12). The federal government is the largest funding source for 36% of biobanks and provided some amount of funding in the past 5 years to 57% of responding biobanks. The larger organization is the largest current source for 30% of biobanks and provided some amount of funding for 59% of biobanks within the past 5 years. Fees for services (primary for 11% and within-5-year source for 44%) and funding from individuals or foundations (primary for 10% and within-5-year source for 40%) were also common. In a separate question, we asked biobanks that are not part of government agencies but nonetheless receive government funding to indicate the extent to which they are dependent on government funding to maintain operation. Among these, 34% are completely dependent, 36% are mostly dependent, 22% are somewhat dependent, and only 8% are not at all dependent on government funding.

**Table 11:** Largest current source of funding

	n	%
Federal government	158	36
The larger organization biobank is a part of	133	30
Fees for services	49	11
Individuals or foundations	43	10
State government	11	2
Clinical and Translational Science Award	11	2
Sale of specimens	10	2
Other	10	2
Sale of other products	9	2
The network to which biobank belongs	5	1
None	4	1
Total	443	100

**Table 12:** Funding sources in past 5 years (check all that apply)

**Percentage of biobanks which received any funding from**...	n	%
The larger organization biobank is part of	269	59
Federal government	260	57
Fees for services	200	44
Individuals or foundations	182	40
Sale of specimens	68	15
State government	53	12
Clinical and Translational Science Award	47	10
The network to which biobank belongs	33	7
Sale of other products	30	7
Interest or dividends	15	3
Licensing technologies	14	3
Local government	5	1

Taken together, these data on embeddedness and multiple affiliations demonstrate the inadequacy of simple classifications of biobanks as 'government, academia, or industry.' For example, the approximately 300 biobanks that identify themselves as 'academic' have essentially the same degree of diversity in organizational features (such as size of collection and sources of acquisition) as the full set of responding biobanks (data not shown).

### Market context and sustainability

To learn about the 'market' in which biobanks operate, we asked respondents whether their biobank is in competition with other biobanks to provide specimens and data to researchers. Only 14% said yes. Of these 57 biobanks, only 4 said there is 'a great deal' of competition; 29 (51%) said there is a moderate amount of competition, and 24 (42%) said there is very little competition. Overall, then, only 33 (8%) of responding biobanks reported that they experience a great deal or a moderate amount of competition. For-profit biobanks are significantly more likely to report being in competition than others (61% versus 12%, *P *< 0.001). It would appear that most biobanks fill a particular 'niche' within their larger organization or research area and generally do not seem concerned about losing their 'market.' This sentiment is echoed in responses to questions about demand for biobank products and services, where 51% of respondents report that demand for their bank's services has increased in the past 2 years. Only 6% said demand decreased, and 43% said it stayed about the same.

While concerns about competition and demand were not substantial, many respondents did express concern about sustainability, typically linked with financial needs. Forty-one percent consider this a major concern, 31% a moderate concern, and 20% a minor concern; only 9% report it is not at all a concern. As might be expected, running out of funding is significantly less likely to be a major or moderate concern for biobanks which rely on sales (of specimens or other products) and fees for services as their primary funding source than it is for others (58% versus 74%, *P *< 0.01). A final marketplace concern for some biobanks is the underutilization of their specimen collections. Thirteen percent report that this is a major concern, 28% say it is a moderate concern, for another 28% it is a minor concern, and 31% said it is not at all a concern.

## Conclusions

In this paper we describe both the process by which we conducted a national U.S. biobank survey and results from that survey. As the first survey of its kind in the U.S., our study has inevitable limitations, particularly in attempting to characterize such a diverse universe of organizations. Given the lack of a comprehensive registry of biobanks from which to sample, our survey cannot claim statistical inference to all U.S. biobanks. However, our systematic, multi-faceted searches for online presence produced a reasonable approximation of all biobanks in existence in the U.S. at this time. We achieved a high survey response rate, but we cannot speak to whether our results would have been different had the other 28% of banks responded. The only significant bias in survey response was whether we had established contact with a potential respondent; this was not, however, related to the biobank's URL domain. (The various URL domains were represented in appropriate proportions among our responding biobanks.) Finally, we document that over half of our respondent banks were established since 2001. While we believe this reflects a burgeoning industry, it does not take into account survival bias. There were undoubtedly some biobanks that were in existence before 2001 but did not survive to be included in our list.

Despite these limitations, the documentation of tremendous diversity in the origins, collections, organizational features, and market contexts of biobanks in the U.S. is important. This diversity raises questions about the utility of the simple classification schemes employed in the past. Our results strongly suggest that a multi-dimensional classification scheme is needed that will enable nuanced attention to the policies and practices that biobanks employ to carry out their work.

Biobanks are not a new phenomenon. However, the steep rate of increase in establishment in the last decade is a sign of rapid change, and likely also contributes to the organizational diversity we document. Undoubtedly, some of this increase may be attributed to advances in genomics and bioinformatics and increased emphasis on translational research, all of which stimulate the demand for stored specimens and associated data. In fact, our survey results indicate that the majority of biobanks are storing specimens that can be used for genetic or genomic research, which raises particular concerns regarding privacy and identifiability [41]. Our prior work on genetic researchers' perspectives documents their reluctance to discard samples [42], and preliminary data analysis from our case studies and open-ended comments from our survey confirm this belief in the tremendous value of research specimens.

Findings that document highly diverse numbers, types, and sources of specimen collections raise questions regarding how biobanks should be managed and governed. For example, while the large number of banks that collect and retain residual clinical specimens is unsurprising, it is nevertheless important because of recent controversies regarding whether and how consent for such specimens should be obtained [41,43-45]. Perhaps more surprising is our finding that such a large number of biobanks contain pediatric and postmortem specimens in their collections, along with specimens from other sources. While biobanks with exclusively pediatric or postmortem specimens (9% and 2% of our surveyed biobanks, respectively) typically adopt particular guidelines for human subjects protections, it is less clear how these specimens might impact governance in mixed-source collections.

Most biobanks are embedded in one or more other entities. The fact that over one-quarter of surveyed biobanks are part of more than one larger organization creates significant classification problems. Embeddedness makes it harder to understand how policies and practices are enacted, as it may be unclear where the locus of decision-making resides. More research is needed to understand the complexity of biobanks' relationships with other organizations and how these relationships impact their work.

The finding of remarkable diversity in organizational characteristics is typical of an emerging industry in the early and rapidly evolving stages of development [46]. In fact, a striking finding of rapid turnover in this industry was actually a by-product of our work to create the biobank list (for which the majority of biobanks classified as ineligible after correspondence with each biobank's potential respondent was due to the bank no longer being in operation). Organizational theory would predict that new, innovative forms of biobanks will arise and survive based on a variety of both internal and external factors [46], that these factors may be studied to understand organizations' survival strategies [47,48], and that some forms will become institutionalized over time [49,50].

Capturing the full range of organizational configurations will be important moving forward as the market for biobank services is likely to increase in complexity, and possibly in the mixture of interrelated forms such as public-private partnerships, vertical and horizontal networks, and other types of out-sourcing or combined practices. It is difficult to predict the nature and extent of change for biobank organizations. However, given that the majority of respondents in our study expressed concern about funding and underutilization, one scenario is that biobanks will coalesce around a limited number of organizational forms in order to maximize survival, and that those with similar missions may join together to form larger collections, or join networks or consortia to endeavor to stabilize funding and increase utilization. Regulatory agencies, professional associations, or influence exerted by more established biobanks on latecomers might all promote convergence of organizational types. Conversely, a different scenario would predict that biobanks are driven toward increased diversity by rapid changes in technology, customization, and the need to differentiate to address more particularized markets. Innovation might be found more in independent biobank markets where fewer forces may exist to compel banks to be more similar [49]. These are important topics for future research, especially as they relate to utilization of biobank resources and ensuring that biobanks meet their promise as effective vehicles for translational research.

As part of the research enterprise, biobanks, like the researchers who depend on their services and specimens, need guidance informed by knowledge of their practices and challenges. The complex organizational landscape of biobanking requires policies as nuanced as the biobanks themselves, whether those policies address subject protection or privacy, or the advancement of research goals. Given different stakeholders and missions, it is unlikely that one-size policies will fit all biobanks, but attention to organizational diversity is critical for the promotion of appropriate and effective biobank governance.

## Abbreviations

CTSA: Clinical and Translational Science Award; NIH: National Institutes of Health; RAND: Research ANd Development Corporation; UNC: University of North Carolina at Chapel Hill.

## Competing interests

The authors declare that they have no competing interests.

## Authors' contributions

RJC, GEH, CZ, TE, IC, BJW, JPE, and AMD conceived of the study and its design. RJC, AGN, IC, GEH, and TE were involved in acquisition of data. RJC, GEH, AGN, JPE, CZ, IC, BW, and TE were involved in data analysis. All authors participated in drafting the manuscript. All authors read and approved the final manuscript.

## Authors' information

Our authors include individuals with expertise in genetics, medicine, and biobank management (JPE), organizational studies (BJW, CRZ), survey research methodology (TE, IC, RJC), law (AMD), and social science studies of ethical, legal, and social aspects of genetics and genomics (GEH, RJC, AMD).
